# Amygdala and cognitive impairment in cerebral small vessel disease: structural, functional, and metabolic changes

**DOI:** 10.3389/fneur.2024.1398009

**Published:** 2024-07-12

**Authors:** Zhenyu Cheng, Wenying Nie, Junhong Leng, Linfeng Yang, Yuanyuan Wang, Xianglin Li, Lingfei Guo

**Affiliations:** ^1^School of Medical Imaging, Binzhou Medical University, Yantai, Shandong, China; ^2^Jinan Maternity and Child Care Hospital Affiliated to Shandong First Medical University, Jinan, Shandong, China; ^3^Key Laboratory of Endocrine Glucose & Lipids Metabolism and Brain Aging, Ministry of Education, Department of Radiology, Shandong Provincial Hospital Affiliated to Shandong First Medical University, Jinan, Shandong, China

**Keywords:** cerebral small vessel disease, amygdala, magnetic resonance imaging, vascular cognitive impairment, neurodegeneration

## Abstract

Cerebral small vessel disease (CSVD) is a prevalent vascular disorder that has been consistently associated with vascular cognitive impairment (VCI). The diagnosis of CSVD continues to rely on magnetic resonance imaging (MRI). Epidemiological data indicate that the characteristic MRI features of CSVD, including white matter hyperintensity (WMH) and lacunar infarction, are very common among individuals over 40 years of age in community studies. This prevalence poses a significant burden on many low- and middle-income families. The amygdala plays a crucial role in integrating sensory and associative information to regulate emotional cognition. Although many previous studies have linked alterations in the amygdala to various diseases, such as depression, there has been little research on CSVD-associated alterations in the amygdala due to the complexity of CSVD. In this paper, we summarize the various imaging features of CSVD and discuss the correlation between amygdala changes and VCI. We also explore how new neuroimaging methods can assess amygdala changes early, laying a foundation for future comprehensive exploration of the pathogenesis of CSVD.

## 1 Introduction

Vascular cognitive impairment (VCI) is mainly caused by vascular risk factors and cerebrovascular diseases. VCI includes a wide range of cognitive impairments, from mild cognitive impairment to vascular dementia (1). The World Health Organization (WHO) estimates that there are 55 million individuals currently suffering from dementia, and this number is expected to be approximately 80 million by 2030. As average life expectancy increases, the prevalence of age-related diseases has also risen. According to a cross-sectional survey, 21.48% of individuals aged 85 and older experience cognitive impairment, with over 5.77% diagnosed with dementia (2). Vascular cognitive dysfunction is a significant contributor to cognitive decline and can exacerbate Alzheimer’s disease, which brings a great burden to family and society (3). It is a group of heterogeneous brain diseases with multiple symptoms. The onset is often subtle, making early clinical diagnosis difficult. Among all the diseases that cause VCI, CSVD accounts for about 50%–70% and is the primary pathogenic factor of VCI (4). Currently, neuroimaging based on high-resolution magnetic resonance provides important insights into pathogenesis, including the use of diffusion tensor imaging (DTI) and quantitative susceptibility mapping (QSM) to monitor changes in disease development and progression. We reviewed multiple pieces of neuroimaging evidence for CSVD and its association with vascular cognitive dysfunction. Recent studies have shown that disruption of the amygdala circuit affects decision-making and emotional regulation (5, 6). Although the relationship between amygdala changes and VCI in patients with CSVD remains controversial, multimodal imaging combined with clinical data may provide new evidence for the role of the amygdala in the progression of CSVD. Finally, we highlight the profound therapeutic potential of exploring the pathological mechanisms of CSVD and elucidating the role of the amygdala in neuropsychiatric disorders, which may facilitate early intervention for CSVD-associated VCI.

## 2 Cerebral small vessel disease and vascular cognitive impairment

The pathological process of CSVD involves a variety of cerebral blood vessels, including arterioles, capillaries and venules. CSVD may be a latent onset of minor cerebral ischemia, the onset of occult, often lead to clinical missed diagnosis and misdiagnosis. Although the early symptoms of CSVD are mild, the course of the disease is long and progressive, often leading to VCI, disability, depression, and death. It disrupts the frontal lobo-subcortical network, leading to typical subcortical functional impairment, while memory involvement is relatively low and cognitive function is relatively maintained.

The incidence of CSVD is 5–6 times that of clinical stroke and 6–10 times that of macrovascular stroke. Currently, at least 700 million people worldwide suffer from some form of CSVD, which is 6 to 10 times more common than large vessel stroke (7). CSVD accounts for 25% to 30% of stroke cases, 45% of dementia cases, and 70% of vascular dementia cases (8). The incidence of CSVD does not differ by sex, race, or region but increases with age. Epidemiological studies show that the incidence of white matter lesions (WML) is only 5% at age 50, 80% in people over 60, and as high as 100% at age 90 (9). Similarly, the incidence of cerebral microbleeds (CMBs) is about 6% at ages 45 to 50, increasing to 36% at ages 80 to 89 (5). CADASIL (Cerebral Autosomal Dominant Arteriopathy with Subcortical Infarcts and Leukoencephalopathy) is the most common hereditary CSVD, with a prevalence of about 2 to 4 in 100,000 adults (10).

The pathological process of CSVD involves several underlying physiological and pathological mechanisms, such as blood–brain barrier (BBB) injury, small vessel sclerosis, decreased cerebral blood flow, white matter lesions, chronic ischemia, neuroinflammation, and myelin injury. Despite extensive research, the specific role of these mechanisms in the pathogenesis of CSVD remains poorly understood. Recently, increasing evidence has shown that various brain waste clearance dysfunctions are closely related to the pathogenesis and prognosis of CSVD. The glymphatic system (GS) and meningeal lymphatic vessels (mLV) are analogs of the lymphatic system in the central nervous system (CNS) (11). These systems play a key role in regulating cerebrospinal fluid (CSF) and interstitial fluid (ISF) transport, waste removal, and potentially neuroinflammation. In patients with CSVD, BBB function may be impaired even in white matter that appears normal on imaging, and the degree of impairment may increase with the lesion load (12). Worse, if the BCSFB does not adequately compensate for this damage, neurotoxic substances may infiltrate the brain parenchyma and cause neuroinflammation, thereby promoting the progression of CSVD (13). Evidence suggests that these barrier leaks are a possible early pathological event in the development of CSVD-associated brain injury. Therefore, measuring barrier function is beneficial for early screening and assessing the status of CSVD. Although the relationship between barrier dysfunction and the progression of CSVD is well established, comprehensive intracerebral evaluation studies of BBB and BCSFB leakage are lacking. Establishing advanced neuroimaging methods to directly evaluate their clearance efficiency may contribute to the early diagnosis of CSVD, and improving this clearance function may be an important goal of therapeutic strategies.

## 3 Change of structural and network in amygdala

### 3.1 Change of structural in amygdala

How amygdala structure changes are associated with cognitive decline has long been a mystery. A considerable number of neuroimaging studies has established amygdala volume loss in the CSVD group compared to healthy controls (14, 15). In recent years, with the development of advanced surface mapping technology, more accurate segmentation and measurement of amygdala subregions can be performed, such as FMRIB’s Software Library (FSL) or FreeSurfer. This offers significant convenience for assessing structural alterations in the amygdala. With the help of these automatic segmentation tools, it has been determined that amygdala atrophy in CSVD patients is a common phenomenon rather than an exception.

**Figure 1:**
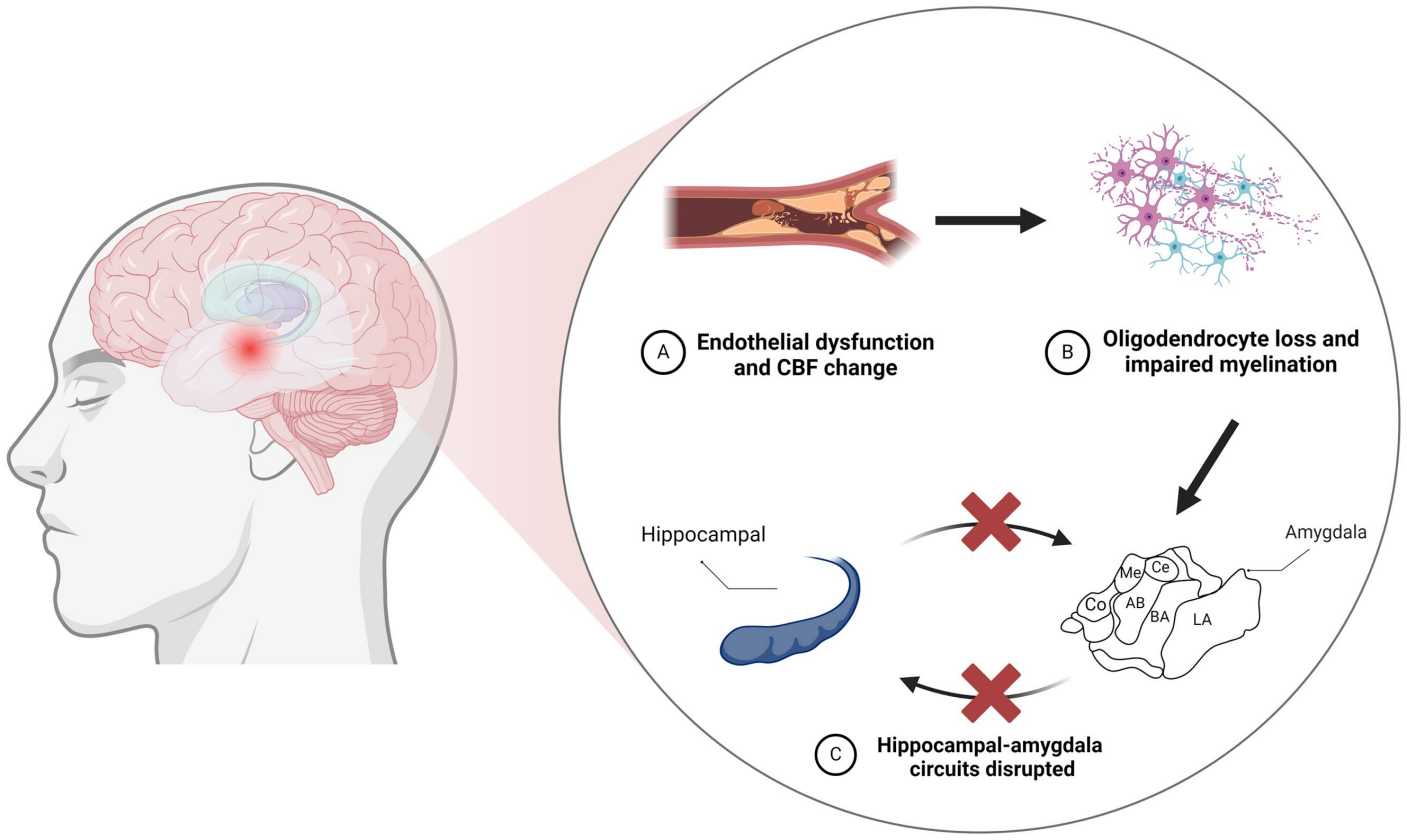
Neurodegeneration resulting from vascular injury leads to disruption of the amygdaloid-hippocampal circuit. **(A)** CBF changes resulting from vascular endothelial cell injury. **(B)** Vascular endothelial cell injury contributes to oligodendrocyte loss and impaired myelination. **(C)** The amygdala acts as a core hub in hippocampus-amygdala circuits, and damage to the amygdala can disrupt these connections. CBF, cerebral blood flow; LA, lateral nucleus; BA, basal nucleus; AB, accessory basal; Co, cortical; Me, medial; Ce, central.

However, most existing research treats the amygdala as a whole structure, leading to a primary focus on changes in total amygdala volume while ignoring the impact of volume changes in subregions on cognition. With the shift from manual to atlas-based automatic segmentation for brain structure volume calculations, we now have the opportunity to explore investigations at the subregion level in more depth (16). As expected, the link between amygdala subregion volume and cognitive impairment was confirmed. Ischemic stroke, a common manifestation of CSVD, is associated with cognitive impairment. A large cohort study from Sydney found that ischemic stroke patients with cognitive impairment had smaller amygdalae compared to those without cognitive impairment. Additionally, the study found that right amygdala volume was negatively associated with visual learning ability. The study also found that a smaller amygdala may increase the risk of depression. Although the link between CSVD and depression is not clear, the atrophy of the amygdala caused by CSVD may increase the risk of depression in patients (17). Population-based studies, such as those by Wei et al., have provided statistical shape analysis of the amygdala in patients with mild cognitive impairment (MCI) compared to healthy controls using multiple Chinese MRI datasets. Their findings highlight a significant reduction in amygdala volume, consistent with a pattern of HC > MCI > AD. Further shape analysis highlighted the presence of numerous region-specific atrophies in all amygdala subregions, with the most severe atrophy confined to the central region, including the basolateral and basomedial subnuclei (18). Importantly, the basolateral amygdala (BLA) projects extensively into the frontal lobe, hippocampus, and sensory cortex, which are components of emotional and cognitive processing. This suggests that deterioration of the BLA may impair cognitive functions that rely on these connections, such as emotion regulation, learning, and memory (Figure 1). Additionally, clinical studies have confirmed that patients with CSVD show the most severe involvement in the basolateral, ventral, and medial amygdala, with local tissue volume reduced by more than 30%. Atrophy was also observed in the central, cortical, and medial nuclei of affected patients (19). These structural changes may account for the association between structural damage to the amygdala subregions and cognitive impairment in CSVD patients, but the specific causal relationship still needs further study.

Increasing evidence suggests that endothelial dysfunction may serve as a trigger for the pathologic changes seen in CSVD, leading to oligodendrocyte loss and consequent myelin impairment (20, 21). The relation of vascular and structural changes in the amygdala should not be underestimated, as neuroimaging studies have demonstrated the downstream impact of these CSVD-related pathological alterations on the amygdala (22). Population-based studies have also established a link between amygdala atrophy and an elevated risk of dementia. For instance, a study integrating brain neuroimaging with prospective cohort data showed that a lower ratio of gray matter volume to total brain volume (GMV/TBV) within the amygdala was linked to a higher risk of disease progression and vascular dementia (VD). Individuals with lower amygdala GMV/TBV ratios faced a higher future risk of dementia (23). Voxel-based morphometry (VBM) studies have further correlated gray matter volume (GMV) and white matter volume (WMV) with cerebral microbleeds (CMBs) in CSVD (24). Their data further suggest that gray matter atrophy in medial temporal lobe structures could represent an early indicator of cognitive impairment in CSVD (25). This evidence indicates that atrophy in key regions, including the medial temporal lobe, hippocampus, and amygdala, substantially predicts the risk of cognitive impairment and dementia.

In summary, the accumulated evidence from structural MRI suggests that atrophy of the basolateral nucleus might disrupt the cortical connections of the amygdala involved in emotional and cognitive processing. This lends further support to the models proposing the basolateral amygdala as a pivotal target of CSVD pathology. With the help of these dynamic structural changes and their association with the amygdala and its network, we may identify new biomarkers and enhance our understanding of the pathophysiological bases of cognitive impairment in CSVD.

### 3.2 Change of structural network in amygdala

Previous studies have confirmed that the amygdala is connected to other critical brain regions through white matter tracts, such as the uncinate fasciculus (UF). DTI studies have revealed damage in these white matter connections between the amygdala and other regions in CSVD^26^. In particular, the UF, which links the amygdala with the prefrontal cortex and hippocampus, exhibits reduced fractional anisotropy and structural connectivity between the amygdala and anterior cingulate cortex (26). The cingulum bundle, another tract connecting the amygdala and hippocampus, also shows impaired integrity in CSVD. This finding aligns with observed cortical thinning in frontal areas sharing connections with the amygdala (27). Surface-based morphometric analysis indicates that cortical atrophy concentrically affects the bilateral medial orbitofrontal, rostral middle frontal, superior frontal, and frontal pole regions in CSVD patients compared to controls (28). The magnitude of cortical thinning in the medial orbitofrontal cortex significantly correlates with basolateral amygdala atrophy. Similar relationships were found between amygdala volume loss and parietal cortical thinning affecting the bilateral inferior parietal, supramarginal, and precuneus regions. As the basolateral amygdala maintains extensive projections with frontal, hippocampal, and sensory cortical regions, its deterioration may impair emotional regulation, learning, and memory functions that rely on these connections (29).

Furthermore, longitudinal studies indicate that greater baseline amygdala atrophy predicts more severe future cortical thinning in synaptically connected target regions in patients with subcortical ischemic vascular dementia (17). These findings lend support to models proposing that tau and amyloid pathologies propagate transsynaptically, initiating within the amygdala before disseminating to interconnected cortical target regions. This transneuronal propagation model has been verified by proteomic data (30). The observed pattern of structural changes first affecting the amygdala and then encroaching on interconnected cortical areas aligns with the sequence expected from this proposed transsynaptic mechanism (31). Further research combining multiple structural MRI modalities, graph theory network analysis, and longitudinal data will provide greater insights into the precise spatiotemporal sequence of gray and white matter deterioration centered on the amygdala. By clarifying these dynamic structural changes and their association with the amygdala and its network, we may uncover novel biomarkers and refine our understanding of the pathophysiological underpinnings of cognitive impairment in CSVD.

## 4 Association between amygdala changes and cognition in CSVD

Functional MRI studies have unveiled disrupted connectivity patterns involving the amygdala in CSVD patients. Compared to healthy controls, these individuals exhibit diminished functional connections between the amygdala and specific prefrontal cortical regions, namely the anterior cingulate cortex and the medial prefrontal cortex (32). Such disruptions within front-limbic circuits are likely contributors to the deficits in emotional regulation and processing frequently occur in CSVD patients (32, 33). Additionally, resting-state fMRI has highlighted impaired connectivity between the amygdala and key nodes of the default mode network, including the posterior cingulate cortex and precuneus (34). This finding suggests that the aberrant functional interactions with cortical areas involved in internally focused cognition may contribute to CSVD-related impairments (35). Seed-based analysis further reveals that the amygdala’s connectivity with the salience network is particularly compromised (29). Longitudinal research suggests that initial levels of amygdala-prefrontal cortex connectivity might predict future cognitive decline. Therefore, there is a pressing need for research employing parcellation and dynamic functional connectivity analysis to better understand changes in amygdala subregion connectivity and the temporal patterns of amygdala interactions throughout CSVD progression.

Recent studies have extended our understanding of the amygdala’s role, providing evidence of its involvement in memory encoding beyond emotional items. Neural analyses indicate that the amygdala creates unique memory imprints for different stimuli, underscoring its broader function in memory beyond emotional processing (36). Beyond its direct role in memory, the amygdala’s connections with broader brain networks are critical for coordinating information processing. These results indicate a compromised role of the amygdala as a hub in the brain’s network and a breakdown in the community coordination essential for emotional and memory processes. Structural modeling has demonstrated that this network disruption mediates the relationship between white matter injury and cognitive deficits in CSVD (37, 38). It is critical to keep in mind that such alterations are not isolated but are widespread across various cognitively impaired groups, suggesting that the observed amygdala network changes are a common feature of the disease. Moreover, further analysis using multivariate methods has revealed that characteristics of the amygdala network can significantly predict executive and memory dysfunction in CSVD (39). These convergent findings from multiple analytical methods lend robust support to the notion that disruptions in the amygdala’s network connectivity are a crucial factor underlying cognitive impairment in CSVD (29, 40).

Overall, rs-fMRI evidence implicates the amygdala in the modulation of global cognitive processes and neuropsychiatric states in CSVD patients. The atrophy of the amygdala and the disruption of its connections to critical cortical networks are likely to impair the normal functioning of these brain circuits (41). The disruption connectivity of the amygdala could be a significant factor affecting cognitive and contributing to psychiatric symptoms in patient group.

## 5 Discussion

### 5.1 Amygdala damage in CSVD

In line with decades of findings from animal studies, the amygdala plays a critical role in our experience of life. Multiple previous studies of amygdala damage have demonstrated that stability of our cognition and fear depends on this specific structure, and that when this structure of widespread connection and widespread influence is missing, our experience, perception, or recognition of fear is impaired. These therapeutic possibilities are bolstered by the wealth of data from basic research showing that basolateral amygdala atrophy with both structural and functional connectivity disruptions may play an important role in the process of CSVD-related cognitive decline. Evidence of iron accumulation, perfusion abnormalities, metabolic dysfunction, and atypical activation patterns further supports the amygdala’s involvement. Although correlational analyses and longitudinal studies tentatively support the hypothesis of amygdala degeneration leading to transsynaptic neurodegeneration, more research is needed to definitively establish this link. As research data accumulate, the amygdala’s susceptibility to CSVD and its connection to cognitive deterioration warrant further examination (42–44).

### 5.2 Application of advanced imaging techniques in amygdala assessment

**Figure 2:**
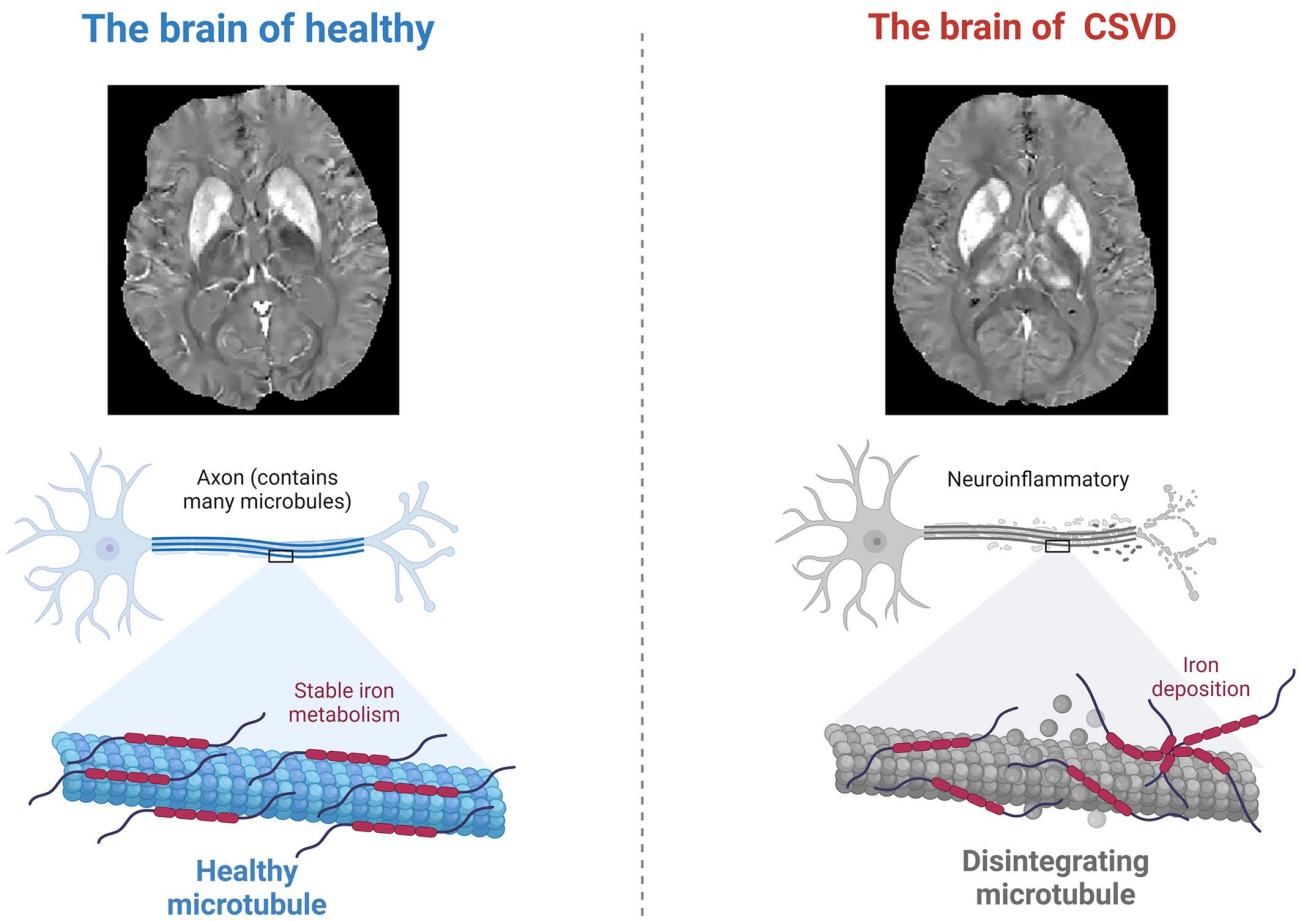
Neuroinflammation and altered iron metabolism. During the neurodegenerative changes caused by CSVD, the process of iron deposition in the brain may cause neuronal damage through oxidative stress.

Although the assessment of amygdala volume by neuroimaging is well established, recent studies have found that iron deposition has sufficient potential as an early sign of neuronal damage. For instance, the accumulation of iron in the brain is thought to contribute to oxidative stress, disrupt mitochondrial function, and initiate alpha-synuclein aggregation, leading to neuronal damage and loss. A prospective cohort study of patients with cerebral small vessel disease and cerebral microbleeds (CSVD-CMBs) suggests that a higher iron deposition load in brain microbleed lesions may serve as a quantitative imaging marker of cognitive decline in CSVD patients. The study computed brain QSM maps from multi-echo gradient echo (mGRE) data using morphology-enabled dipole inversion with automatic uniform cerebrospinal fluid zero reference algorithm (MEDI+0), the latest imaging technology that allows for more accurate assessment of iron deposits in the brain (45). Intriguingly, this heightened iron accumulation has been found to inversely correlate with cognitive performance, as evaluated by the Montreal Cognitive Assessment (MoCA) (46, 47). Similarly, QSM has identified a potential link between iron deposition in the amygdala and cognitive impairment in CSVD, further underscoring the significance of this imaging technique in understanding neurodegenerative processes. In patients with subcortical vascular dementia, which is a variant of CSVD, studies have reported higher magnetic susceptibility in the amygdala, suggesting increased iron content, compared to healthy individuals. Significantly, these elevated iron levels have been found to correlate with the severity of memory impairment (Figure 2).

### 5.3 Future directions and research outlook

Previous research has established the amygdala’s significant role in cognitive impairment associated with CSVD. Future studies ought to understand the temporal evolution of changes in the amygdala and how these alterations affect interconnected cortical areas. Such research is crucial for deepening our understanding of the disease’s progression. Combining multimodal imaging with cognitive assessments and possibly integrating interventional approaches like real-time fMRI neurofeedback could shed light on causal relationships and inform therapeutic strategies. Additionally, molecular imaging may offer insights into the accumulation of neuropathological markers such as amyloid and tau in relation to CSVD. There are already teams using innovative machine learning segmentation models to study the brain’s nuclear subregions, even at the molecular level (48–51). By expanding the scope of the study beyond the connections of the amygdala to include a more fine-grained view of its subregions, we can expect to improve diagnostic criteria and develop specialized interventions to prevent the neurodegenerative processes of CSVD.

## Author contributions

ZC: Writing – original draft. WN: Writing – review & editing. JL: Investigation, Writing – review & editing. LY: Data curation, Writing – review & editing. YW: Supervision, Writing – review & editing. XL: Supervision, Writing – review & editing. LG: Writing – review & editing.
